# Physiological and Pharmacological Modulation of the Embryonic Skeletal Muscle Calcium Channel Splice Variant Ca_V_1.1e

**DOI:** 10.1016/j.bpj.2015.01.026

**Published:** 2015-03-10

**Authors:** Bruno Benedetti, Petronel Tuluc, Vincenzo Mastrolia, Clemens Dlaska, Bernhard E. Flucher

**Affiliations:** 1 Department of Physiology and Medical Physics, Medical University Innsbruck, Innsbruck, Austria; 2 Pharmacology and Toxicology, Institute of Pharmacy, University of Innsbruck, Innsbruck, Austria

## Abstract

Ca_V_1.1e is the voltage-gated calcium channel splice variant of embryonic skeletal muscle. It differs from the adult Ca_V_1.1a splice variant by the exclusion of exon 29 coding for 19 amino acids in the extracellular loop connecting transmembrane domains IVS3 and IVS4. Like the adult splice variant Ca_V_1.1a, the embryonic Ca_V_1.1e variant functions as voltage sensor in excitation-contraction coupling, but unlike Ca_V_1.1a it also conducts sizable calcium currents. Consequently, physiological or pharmacological modulation of calcium currents may have a greater impact in Ca_V_1.1e expressing muscle cells. Here, we analyzed the effects of L-type current modulators on whole-cell current properties in dysgenic (Ca_V_1.1-null) myotubes reconstituted with either Ca_V_1.1a or Ca_V_1.1e. Furthermore, we examined the physiological current modulation by interactions with the ryanodine receptor using a chimeric Ca_V_1.1e construct in which the cytoplasmic II-III loop, essential for skeletal muscle excitation-contraction coupling, has been replaced with the corresponding but nonfunctional loop from the Musca channel. Whereas the equivalent substitution in Ca_V_1.1a had abolished the calcium currents, substitution of the II-III loop in Ca_V_1.1e did not significantly reduce current amplitudes. This indicates that Ca_V_1.1e is not subject to retrograde coupling with the ryanodine receptor and that the retrograde coupling mechanism in Ca_V_1.1a operates by counteracting the limiting effects of exon 29 inclusion on the current amplitude. Pharmacologically, Ca_V_1.1e behaves like other L-type calcium channels. Its currents are substantially increased by the calcium channel agonist Bay K 8644 and inhibited by the calcium channel blocker nifedipine in a dose-dependent manner. With an IC50 of 0.37 *μ*M for current inhibition by nifedipine, Ca_V_1.1e is a potential drug target for the treatment of myotonic dystrophy. It might block the excessive calcium influx resulting from the aberrant expression of the embryonic splice variant Ca_V_1.1e in the skeletal muscles of myotonic dystrophy patients.

## Introduction

The voltage-gated calcium channel isoform Ca_V_1.1 functions as the voltage sensor in skeletal muscle excitation-contraction (EC) coupling. This primary function of Ca_V_1.1 depends on its physical interaction with the skeletal muscle ryanodine receptor (RyR1), but does not require calcium influx through the voltage-gated calcium channel itself. In fact, the classical adult Ca_V_1.1a splice variant requires ∼30 mV stronger depolarization to activate its L-type calcium current (LTCC) than to activate EC coupling (1). Moreover, Ca_V_1.1a currents are small and slowly activating. Of importance, expression of these currents depends on the interaction of Ca_V_1.1a with the RyR1. In the absence of RyR1—either in dyspedic (RyR1-null) myotubes or when Ca_V_1.1a is expressed in heterologous cells—LTCC are barely detectable (2,3). Thus, in skeletal muscle Ca_V_1.1 and RyR1 functionally interact with each other in two directions: By orthograde coupling the Ca_V_1.1 voltage sensor prompts the gating of the RyR1 calcium release channel in response to membrane depolarization. By retrograde coupling the RyR1 augments the LTCC through Ca_V_1.1a severalfold.

Recently, we identified a splice variant of Ca_V_1.1 (Ca_V_1.1e), which is the dominant Ca_V_1.1 expressed in embryonic skeletal muscle (4). It is generated by skipping exon 29 and results in a channel lacking 19 amino acids in the extracellular loop connecting transmembrane segments S3 and S4 in the fourth homologous repeat. Like the full-length Ca_V_1.1a, Ca_V_1.1e is incorporated into skeletal muscle triads and can activate EC coupling independent of calcium conduction through the channel (5). However, in contrast to the poor gating properties of the adult Ca_V_1.1a channel, the embryonic Ca_V_1.1e splice variant conducts calcium currents that are 6–8 times larger than those of Ca_V_1.1a, and activation of its current and of EC coupling occur at the same membrane potentials. Thus, the embryonic Ca_V_1.1e splice variant combines skeletal muscle-type EC coupling properties with cardiac muscle-like calcium currents. However, currently it is not known whether Ca_V_1.1e also supports retrograde coupling.

Both, orthograde and retrograde coupling critically depend on a specific sequence in the cytoplasmic loop connecting homologous repeats II and III of skeletal muscle Ca_V_1.1 channels. When the critical II-III loop sequences were replaced by corresponding sequences of the cardiac Ca_V_1.2 isoform (6) or more distantly related Ca_V_1 channels, skeletal muscles EC coupling, as well as retrograde coupling failed (7,8). Because Ca_V_1.1e contains the critical II-III loop sequences and displays normal orthograde coupling, it is expected to also show retrograde coupling.

Continued expression of the embryonic Ca_V_1.1e splice variant in mature muscles was shown to correlate with the degree of muscle weakness in patients with myotonic dystrophy (9). Furthermore, forced skipping of exon 29 aggravated the disease phenotype in a mouse model of myotonic dystrophy, suggesting that expression of the calcium-conducting Ca_V_1.1e variant in adult muscle may contribute to the etiology of myotonic dystrophy phenotype (9,10). Therefore, use of clinically approved calcium antagonists may be of therapeutic use in the treatment of myotonic dystrophy, provided the Ca_V_1.1e splice variant is sensitive to L-type channel blockers.

The family of Ca_V_1 channels is sensitive to dihydropyridines (DHPs). Bay K 8644 augments LTCC by shifting the fraction of total channel open events markedly to the long opening mode without altering the rate of activation (11). Channel blockers, like nifedipine, reduce the current density by stabilizing the inactivated state of the channel (12). Because skipping of exon 29 does not affect the sequences known to bind DHPs, Ca_V_1.1e would be expected to show DHP sensitivity. On the other hand, because Bay K 8644 as well as deletion of exon 29 enhance the current density with little effect on the rate of activation, it is possible that Bay K 8644 acts on the same downstream molecular mechanism that is altered in Ca_V_1.1e. In this case a reduced sensitivity of Ca_V_1.1e to Bay K 8644 would be expected.

Here, we reconstituted dysgenic myotubes with either the Ca_V_1.1e or the Ca_V_1.1a splice variant to characterize their sensitivity to DHPs, or with a Ca_V_1.1e chimera in which the II-III loop has been replaced with the corresponding sequences of the Musca Ca_V_1 channel to examine its interactions with RyR1. Our pharmacological analysis shows that Ca_V_1.1e LTCC can be blocked by nifedipine or further increased by Bay K 8644. However, replacing the II-III loop abolished skeletal muscle-type EC coupling without reducing the LTCC amplitude, indicating that wild-type Ca_V_1.1e currents are not augmented by retrograde coupling with the RyR1. These findings suggest that the inclusion of exon 29 in the adult Ca_V_1.1a splice variant applies a brake on voltage-dependent current activation that is partially alleviated by retrograde coupling with the RyR1. In the absence of this brake in Ca_V_1.1e retrograde coupling is ineffective.

## Materials and Methods

### Expression plasmids

Ca_V_1.1a: GFP-*α*_1S_ (13); Ca_V_1.1e: GFP-*α*_1S_-ΔE29 (5). Ca_V_1.1e-SkLM: GFP-SkLM-ΔE29 was generated by excising a 1777 bp fragment containing the second half of repeat III and repeat IV using BglII and XhoI enzymes from GFP-*α*_1S_-ΔE29 plasmid (5) and ligating it into the homologous region of the GFP-*α*_1S_-SKLM channel previously described (7).

### Muscle cell culture and transfection

Myotubes of the homozygous dysgenic (mdg/mdg) cell line GLT were cultured as previously described (14). At the onset of myoblast fusion, GLT cell cultures were transfected with plasmids coding for the calcium channel subunits using FuGeneHD transfection reagent (Promega, Mannheim, Germany) according to the manufacturer’s instructions. A total of 1 *μ*g of plasmid DNA was used per 30 mm culture dish.

### Electrophysiology, fluorescence calcium recording, and pharmacology

Patch pipettes (borosilicate glass, Harvard Apparatus, Holliston, MA) had resistance of 1.5–3 MΩ when filled with (mM) 145 Cs-aspartate, 2 MgCl_2_, 10 HEPES, 0.1 Cs-EGTA, 2 Mg-ATP, and 0.2 Fluo-4 when calcium transients were recorded in parallel to calcium influx (pH 7.4 with CsOH). The extracellular bath solution contained (mM) 10 CaCl2, 145 tetraethylammonium chloride, 10 HEPES (pH 7.4 with tetraethylammonium hydroxide). To block the inward calcium currents, 0.5 mM Cd^2+^ and 0.2 mM La^3+^ (Cd^2+^/ La^3+^) were added to the bath solution. All recordings were made with an Axopatch 200A amplifier (Axon Instruments, Foster City, CA). Data acquisition and command potentials were controlled by pClamp software (version 8.0, Axon Instruments). Fluorescent calcium signals were recorded in parallel to patch clamp recordings using PTI photomultipliers (Photon Technology International, Seefield, Germany). The voltage dependence of currents and calcium signals were analyzed as described in (4). DHPs were dissolved in dimethyl sulfoxide (DMSO) at the concentration of 1 mM and diluted in the bath solution at the following concentrations (*μ*M): Bay K 8644: 1, Nifedipine: 0.1, 0.5, 5. When the highest concentration of DMSO (0.5%) was also applied to the bath solution in absence of DHPs, it had scarce effect on the calcium currents (I_Max DMSO_/I_Max control_ = 0.8 ± 0.09) and voltage dependence (−5 ± 1 mV) of CaV1.1e.

### DHP application

The effect of DHPs were tested in voltage-clamp experiments with a multiple-sweep protocol containing 200 ms long pulses from −50 mV to +80 mV in 10 mV increments before and after DHPs application. Between sweeps, the membrane voltage was kept at −80 mV (I_hold_ < 100 pA). To inactivate T-type currents, a 50 ms prepulse (to −50 mV) was applied before each sweep. Series resistance (Rs) was monitored between protocols, rejecting recordings with Rs drift > 20% or with Rs > 12 mΩ. After stable control recordings, the DHP-containing extracellular solution was applied at a flow rate of 1.5–2.5 ml/min, replacing at least twice the chamber volume. In these conditions, after 6 to 8 min of drug application, full drug effect was observed. Tail current amplitude was measured before and after Bay K 8644 application to quantify the changes in open probability (Po), assuming that no significant changes in the number of channels in the cell membrane occurred within 6–8 min of drug application. We have never observed a rundown of Ca_V_1.1a or Ca_V_1.1e in dysgenic myotubes.

### Immunofluorescence and antibodies

Immunofluorescence analysis was performed as described (15) using the following antibodies: rabbit polyclonal anti-GFP (1:4,000; Molecular Probes, Eugene, OR); mouse monoclonal anti-RyR (34-C; 1:1000; Alexis Biochemicals, Lausen, Switzerland); secondary goat-antimouse Alexa-594 and goat-antirabbit Alexa-488 (1:4,000; Molecular Probes). Images were captured on a Zeiss Axiophot microscope with a cooled charge-coupled device camera and METAVUE image-processing software (Universal Imaging, West Chester, PA).

## Results

### Ca_V_1.1e currents can be augmented by the L-type calcium channel agonist Bay K 8644

**Figure 1:**
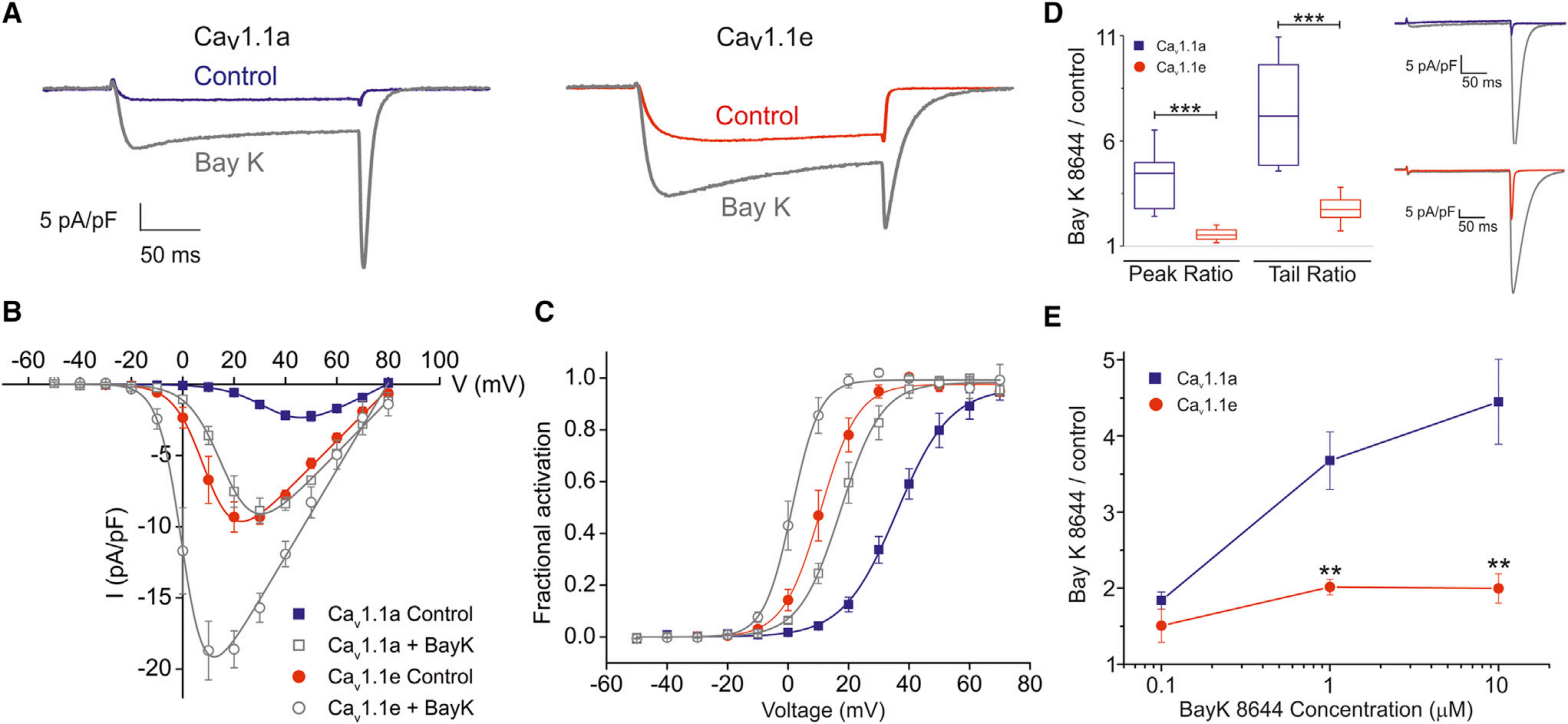
Bay K 8644 sensitivity of the embryonic Ca_V_1.1e and adult Ca_V_1.1a calcium channel splice variants in skeletal myotubes. (*A*) Representative patch clamp recordings before and after Bay K 8644 (*gray*) application from dysgenic myotubes reconstituted with either Ca_V_1.1a (*blue*) or Ca_V_1.1e (*red*). Currents (I_Max_) elicited by depolarizing pulses to peak current potential (V_Max_), charge carrier 10 mM calcium. (*B* and *C*) I/V curves and fractional activation plots indicate that 10 *μ*M Bay K 8644 augments the current densities and left-shifts the voltage-dependence of activation in both Ca_V_1.1a (*blue*) and Ca_V_1.1e (*red*). (*D*) Box plots (*left*) of the ratio between peak currents before and after application of 10 *μ*M Bay K 8644 recorded at +40 mV test pulses, and of the ratio between tail currents recorded at −80 mV following a +60 mV test pulse. Both ratios are significantly larger in Cav1.1a than in Cav1.1e (*p* = 0.002). Representative tail currents (*right*) of Cav1.1a (*blue*) and Cav1.1e (*red*) before and after Bay K 8644 application (*gray*). (*E*) Relative increase in I_Max_ of Ca_V_1.1a (*blue*) or Ca_V_1.1e (*red*) stimulated by three different Bay K 8644 concentrations. The effect of Bay K 8644 is much larger and concentration dependent in the small currents of Cav1.1a compared to the large Cav1.1e currents. To see this figure in color, go online.

Dysgenic myotubes lack the skeletal muscle calcium channel Ca_V_1.1 and consequently also fail to exhibit LTCC and EC coupling. Both functions can readily be reconstituted by transfection of dysgenic myotubes with Ca_V_1.1 expression plasmids. First, we transfected dysgenic myotubes cultured from the GLT cell line (14) with plasmids encoding either the adult Ca_V_1.1a (GFP-*α*_1S_) or the embryonic Ca_V_1.1e (GFP-*α*_1S_-ΔE29) splice variants. Whole-cell voltage-clamp analysis of myotubes expressing one or the other splice variant revealed calcium currents with greatly distinct properties (Fig. 1). As previously shown (4) Ca_V_1.1a currents were small (−2.0 ± 0.2 pA/pF) and reached half-maximal activation at +35.1 ± 1.7 mV (Table 1; V1/2), whereas Ca_V_1.1e currents had five times larger current amplitudes (−10.4 ± 0.5 pA/pF) and a half-maximal activation at +6.4 ± 1.8 mV. During the 200 ms test pulse both currents showed little inactivation. Previously, we demonstrated that the different calcium current amplitudes are due to different biophysical properties of the two Ca_V_1.1 splice variants and not to different expression levels (4).

Because the observed effects of deleting exon 29—increased current amplitude, left-shifted voltage-dependence of activation, but little change in the rate of activation—closely resemble the known effects of the current-enhancing gating modifier Bay K 8644 (11), we wondered whether skipping of exon 29 in Ca_V_1.1e and the drug action might converge on a common downstream gating mechanism, and thus render Ca_V_1.1e insensitive to Bay K 8644. To examine the possible additivity of Bay K 8644 effects, we recorded whole-cell calcium currents of myotubes expressing Ca_V_1.1a or Ca_V_1.1e before and after bath application of Bay K 8644. Fig. 1 *A* shows the expected effects of Bay K 8644 on current properties of myotubes expressing Ca_V_1.1a. After application of the highest dose of Bay K 8644 (10 *μ*M), the peak current amplitude of Cav1.1a was increased more than fourfold (−9.2 ± 0.8 pA/pF). The half-maximal activation was left-shifted by 19.1 ± 1.5 mV (Table 1). Augmentation of the current density was also observed in myotubes transfected with Ca_V_1.1e (Fig. 1, *A*–*C*). After Bay K 8644 application, the average peak current amplitude was increased by approximately twofold (−19.6 ± 1.5 pA/pF). The half-maximal activation was left-shifted by 9.3 ± 2.6 mV. The fractional inactivation measured at the end of the 200 ms test pulse to potential eliciting the maximal current amplitude was the same before and after the application of Bay K 8644 for both Ca_V_1.1a (20.1 ± 0.03%) and Ca_V_1.1e (22 ± 0.1%). Thus, Ca_V_1.1e is sensitive to Bay K 8644 and the effect of exon 29 skipping and that of the channel agonist are partially additive. However, the effects of Bay K 8644 on Cav1.1a and Cav1.1e are quantitatively different. Bay K 8644 increased the maximal current (I_Max_) of Cav1.1a in a dose-dependent manner (Fig. 1, *D* and *E*), at concentrations between 0.1 *μ*M (approximately twofold) and 10 *μ*M (∼4.5-fold). This did not occur for Cav1.1e, where the increase of I_Max_ saturated (approximately twofold) at Bay K 8644 concentrations of 1 *μ*M. The apparently weaker effects of Bay K 8644 on current amplitudes could be partially explained by the much larger leftward shift in the voltage dependence of activation induced by Bay K 8644 on Ca_V_1.1a currents compared to Ca_V_1.1e currents. To quantify the agonistic effect of Bay K 8644 at the same driving force we also examined the relative increase of tail currents in response to Bay K 8644 for both channel variants (Fig. 1 *D*). The ratio of tail current amplitudes before and after application of Bay K 8644 is significantly larger (*p* = 0.0006) for Cav1.1a (7.1 ± 0.9) compared to Cav1.1e (2.7 ± 0.2). Together, these results show that indeed the effects of Bay K 8644 on voltage sensitivity and current amplitude in Ca_V_1.1e are less than half the magnitude of those in Ca_V_1.1a.

### Ca_V_1.1e currents are sensitive to the L-type calcium channel blocker nifedipine

**Figure 2:**
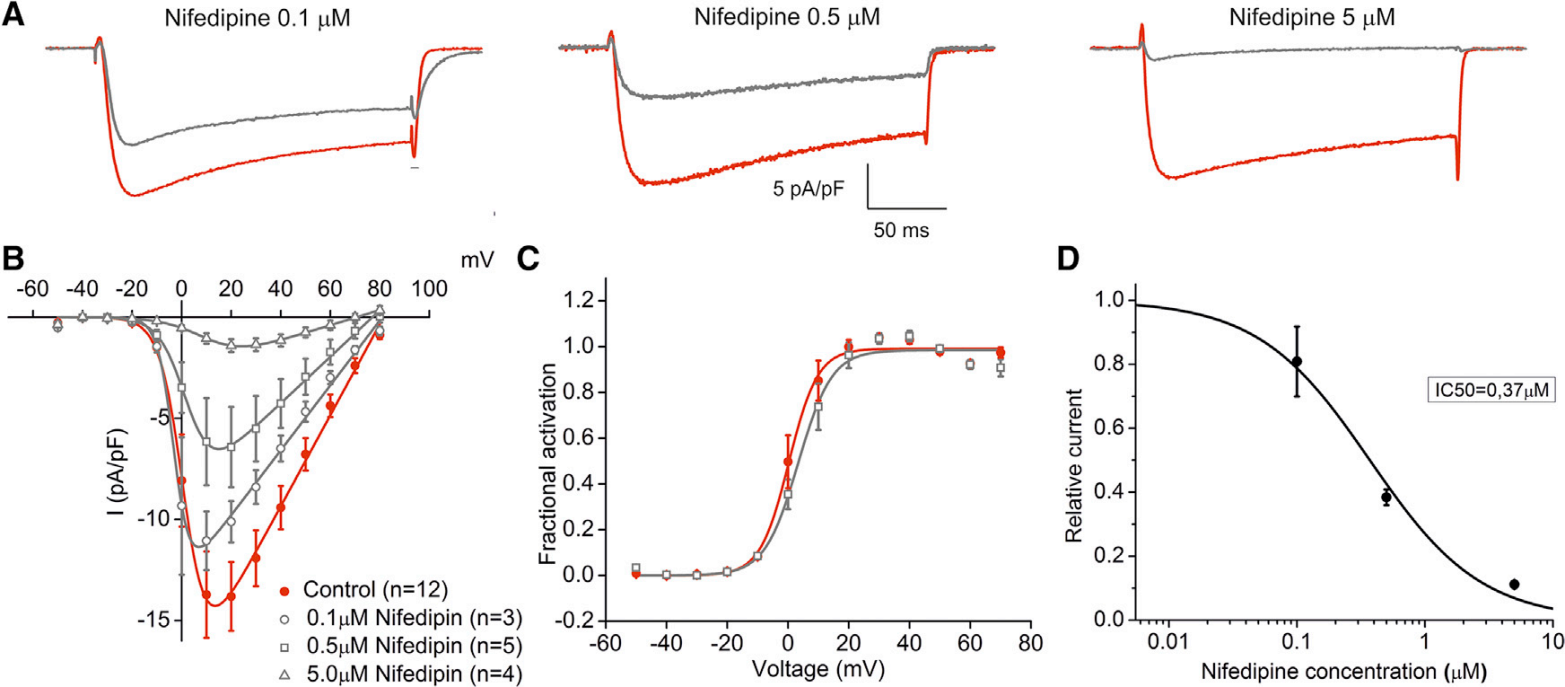
Nifedipine block of Ca_V_1.1e calcium currents. (*A*) Representative calcium current recordings before and after application of 0.1, 0.5, or 5 *μ*M of the LTCC-blocker nifedipine in dysgenic myotubes transfected with GFP-*α*_1S_-ΔE29 (Ca_V_1.1e). (*B*) I/V curves show that nifedipine decreases the current density at all voltages in a dose-dependent manner. (*C*) Nifedipine (0.5 *μ*M) did not affect the voltage-dependence of current activation. (*D*) The dose-response curve of nifedipine-block indicates a IC50 of 0.37 *μ*M. To see this figure in color, go online.

Given our finding that Ca_V_1.1e is sensitive to the agonistic DHP Bay K 8644, it was reasonable to assume that it also would be blocked by antagonistic DHPs. We tested this notion by patch clamp recording of dysgenic myotubes expressing Ca_V_1.1e before and after incubation with 0.1, 0.5, and 5.0 *μ*M of nifedipine for 10 min. The sample current traces in Fig. 2 *A* show that nifedipine at all three concentrations reduced the current amplitude. The highest used concentration of 5 *μ*M nifedipine reduced the peak current density to below 10% (1.4 ± 0.3 pA/pF; Table 2) of that in untreated controls (14.7 ± 2.0 pA/pF) and to 14% compared to controls with the corresponding concentration of the vehicle (0.5% DMSO) (9.7 ± 1.0 pA/pF). The IC50 for the nifedipine block of Ca_V_1.1e currents calculated from the dose-response curve is at 0.37 *μ*M (see Fig. 2 *D*), which is in the same range that has previously been reported for Cav1.2 (16,17) and Cav1.1a (18) in similar cell systems. The IV curves of currents at all three nifedipine concentrations show that the voltage-dependence of activation is scarcely affected by the nifedipine block (Fig. 2 *B*). The −5 mV shift of fractional activation at 0.5 *μ*M nifedipine (Fig. 2 *C*) is likely due to the strong reduction in current amplitude. Thus, the DHP nifedipine inhibits Ca_V_1.1e calcium currents in skeletal myotubes in a concentration-dependent manner, like any other LTCC. Because LTCC through the adult CaV1.1a splice variant are already exceedingly small and functionally irrelevant in physiological EC coupling, we did not attempt to quantify the further reduction of CaV1.1a currents by nifedipine.

### Replacement of II-III loop sequences in Ca_V_1.1e abolishes skeletal muscle-type EC coupling without reducing LTCC density

The data presented thus far show that with respect to its current properties and pharmacological modulation Ca_V_1.1e behaves similar to other L-type calcium channels. However, with respect to EC coupling it exhibits the distinctive skeletal muscle specific attributes: Ca_V_1.1e can activate sarcoplasmic reticulum (SR) calcium release without calcium influx either at depolarizations to the reversal potential, or in the presence of the nonspecific calcium channel blocker Cd^2+^/La^3+^ (4). In the classical/adult Ca_V_1.1a splice variant the molecular interactions underlying the skeletal muscle-specific EC coupling mechanism is also important for the expression of normal, albeit small, calcium currents. Ca_V_1.1a constructs with sequence substitutions in the cytoplasmic II-III loop that prevented EC coupling (i.e., orthograde coupling of the Ca_V_1.1a voltage sensor to the RyR1 calcium release channel) also prevented the RyR1-dependent augmentation of LTCC (i.e., retrograde coupling) (6–8).

**Figure 3:**
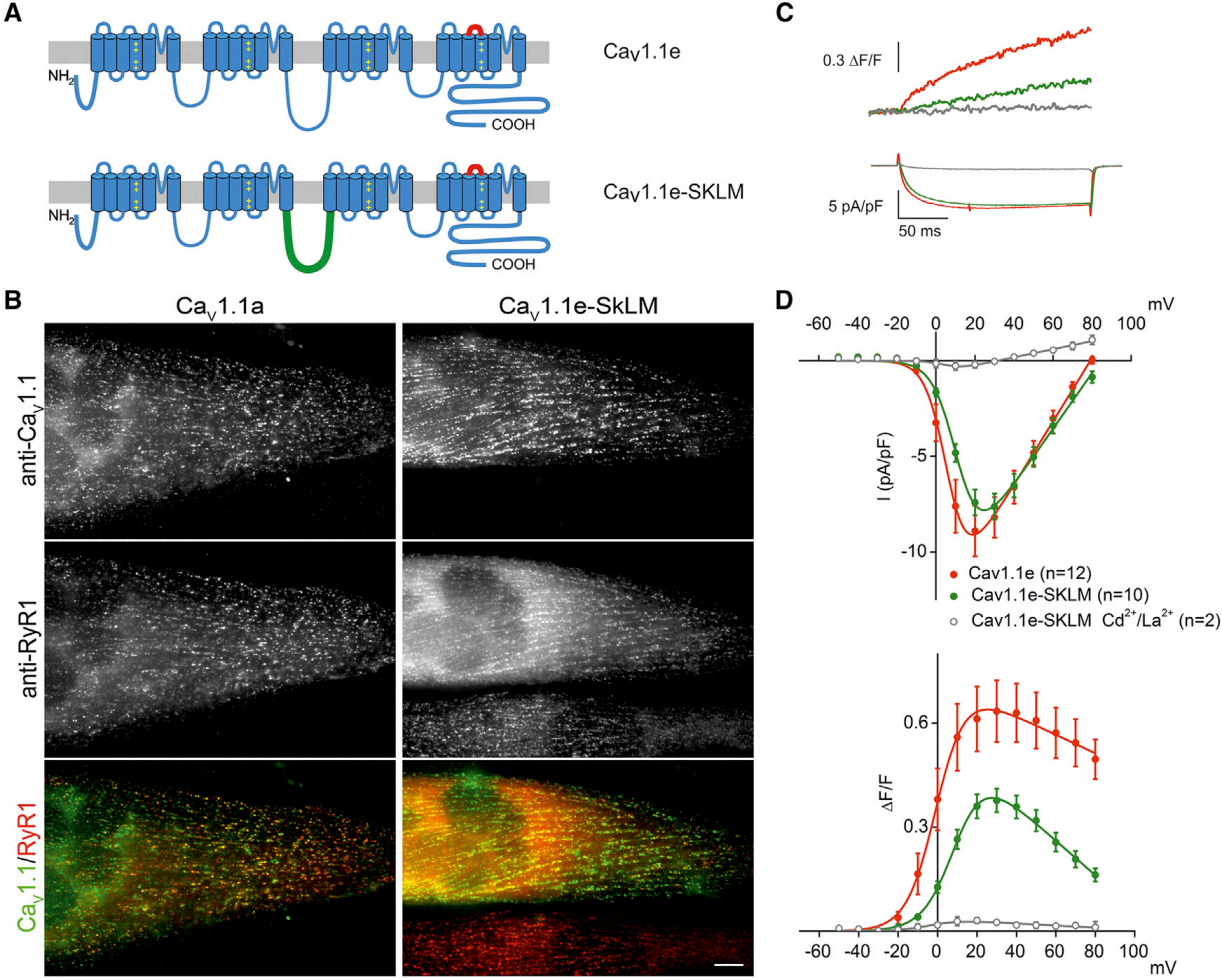
Ca_V_1.1e calcium currents are not augmented by retrograde coupling with the RyR1. (*A*) Domain structure of Ca_V_1.1 depicting the location of the sequence coded by exon 29 (*red*), which is missing in the embryonic Ca_V_1.1e variant, and the II-III loop that has been swapped with the nonfunctional loop of the Musca channel in Ca_V_1.1e-SkLM (*green*). (*B*) Double-immunofluorescence labeling of Ca_V_1.1e variants and the RyR1 in dysgenic myotubes transfected with either Ca_V_1.1e or Ca_V_1.1e-SkLM. Both channels form clusters colocalized with the RyR1. Bar, 10 *μ*m. (*C*) Representative current traces and fluorescence calcium recordings from myotubes expressing Ca_V_1.1e or Ca_V_1.1e-SkLM. (*D*) Voltage-dependence of peak current densities and fluorescent calcium transients. Ca_V_1.1e-SkLM (*green*), compared to Ca_V_1.1e (*red*), expresses similar size calcium currents but decreased calcium transients, which are fully blocked by Cd^2+^/La^3+^ (*gray*). To see this figure in color, go online.

To study whether the expression of the large calcium currents of Ca_V_1.1e in skeletal muscle cells also requires direct interactions with the RyR1, we generated a Ca_V_1.1e in which the II-III loop has been replaced by the corresponding but highly heterologous sequence of the Musca channel (Ca_V_1.1e-SkLM; Fig. 3 *A*). When expressed in dysgenic myotubes Ca_V_1.1e-SkLM was normally incorporated into peripheral junctions and developing triads, as indicated by its colocalization with RyR1 clusters at the cell surface (Fig. 3 *B*). Thus, Ca_V_1.1e-SkLM fulfills the basic structural condition for a functional interaction with the RyR1.

Next, dysgenic myotubes expressing either the wild-type Ca_V_1.1e or Ca_V_1.1e-SkLM were analyzed in parallel with combined whole-cell patch-clamp measurements and fluorometric recordings of the fluorescent calcium indicator fluo-4. Fig. 3 *C* shows representative traces of the cytoplasmic calcium transients (*top*) and calcium currents (*bottom*) at depolarizing steps to the maximally activating voltages. Plotting the voltage-dependence of peak calcium transients and calcium currents (Fig. 3 *D*) shows that calcium transients and calcium currents of Ca_V_1.1e and Ca_V_1.1e-SkLM activate in parallel at the same voltages. Whereas at the reversal potential of +80 mV current densities decline to near zero, Ca_V_1.1e calcium transients are still substantially activated, consistent with calcium influx-independent activation of SR calcium release (i.e., skeletal muscle EC coupling). Calcium currents in myotubes expressing the chimeric Ca_V_1.1e-SkLM channel are very similar in size and voltage-dependence to those of Ca_V_1.1e. However Ca_V_1.1e-SkLM calcium transients are greatly reduced in amplitude compared to those of Ca_V_1.1e and they strongly decline at higher voltages, suggesting a strong dependence on the calcium influx. Application of 0.5 *μ*M Cd^2+^/0.2 *μ*M La^3+^ efficiently blocks the calcium currents at all voltages. In parallel calcium transients are abolished, indicating that the remnant calcium transients observed with Ca_V_1.1e-SkLM are fully dependent on calcium currents. Thus, the small Ca_V_1.1e-SkLM calcium transients arise from calcium influx through the Ca_V_1.1 channel and possibly from calcium-induced calcium release from the SR (i.e., cardiac-type EC coupling (19)). Evidently, the sequence substitution in the II-III loop of the Ca_V_1.1e-SkLM channel has totally abolished skeletal muscle-type EC coupling. This corresponds to what has previously been shown for Ca_V_1.1a-SkLM (6) and indicates that in Ca_V_1.1e-SkLM the direct coupling between the Ca_V_1.1 voltage sensor and the RyR1 has been severed. Nevertheless, the amplitude of its calcium currents was unchanged, demonstrating that Ca_V_1.1e-SkLM’s current size does not depend on II-III loop interactions with the RyR1. Thus, Ca_V_1.1e is not modulated by retrograde coupling.

## Discussion

The skeletal muscle Ca_V_1.1 is unique among the voltage-gated calcium channels in that it can activate SR calcium release independent of calcium influx by direct interactions with the RyR1. In the adult channel variant Ca_V_1.1a this comes at the price of poor current properties. However, the embryonic Ca_V_1.1e splice variant combines both skeletal muscle-type EC coupling properties with perfectly fine current properties. Here, we asked the questions of whether and how Ca_V_1.1e calcium currents can be modulated; physiologically by retrograde interactions with the RyR1 and pharmacologically by DHPs. Our data show that 1) the current-enhancing effect of skipping exon 29 in Ca_V_1.1e precludes further enhancement of the current by retrograde coupling, 2) the Ca_V_1.1e currents can be further augmented by L-type current agonist Bay K 8644, and 3) blocked by L-type current antagonist nifedipine. These findings have important implications for understanding the molecular mechanisms regulating current activation and EC coupling in the skeletal muscle calcium channels, for understanding the function of the embryonic Ca_V_1.1e splice variant in EC coupling, and for assessing its potential as drug target in myotonic dystrophy.

### Ca_V_1.1e is not subject to retrograde coupling

**Figure 4:**
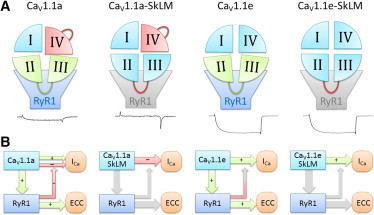
Gating model explaining the effects of exon 29 and the II-III loop-RyR1 interactions on skeletal muscle calcium currents. (*A*) In the adult splice variant Ca_V_1.1a the presence of the extracellular loop encoded by exon 29 impedes channel gating (*red domain IV*). However, interactions of the II-III loop with RyR1 counteract and partially alleviate this inhibition (*green*), enabling small calcium currents in wild-type Ca_V_1.1a. Without this interaction, as in the Ca_V_1.1a-SkLM mutant, exon 29-containing channels conduct virtually no currents. In the absence of exon 29 in the embryonic splice variant Ca_V_1.1e there is no current inhibition by domain IV. Consequently, there is nothing to alleviate by the II-III loop interactions with RyR1 and its substitution in Ca_V_1.1e-SkLM does not alter the current density. (*B*) Our current and published data are best explained by a model according to which retrograde coupling with the RyR1 relieves an inhibitory mechanism that exists in Ca_V_1.1a but not in Ca_V_1.1e. Without coupling to the RyR1 the intrinsic inhibitory mechanism affected by exon 29 prevails in Ca_V_1.1a-SkLM. In the absence of this inhibitory mechanism, as in Ca_V_1.1e and Ca_V_1.1e-SkLM, retrograde coupling is ineffective. To see this figure in color, go online.

When the crucial sequences for Ca_V_1.1-RyR1 interaction in Ca_V_1.1e were eliminated, EC coupling failed but surprisingly the calcium currents were not affected. This finding is unexpected because Ca_V_1.1a and Ca_V_1.1e, which differ only with respect to the inclusion of 19 amino acids in an extracellular loop of Ca_V_1.1a, interact with the RyR1 in the same way, and because earlier experiments with the adult splice variant Ca_V_1.1a demonstrated that retrograde coupling is linked to orthograde coupling (skeletal muscle-type EC coupling) by their dependence on the same critical sequences in the II-III loop (6–8). Based on these studies orthograde and retrograde coupling were viewed as the two sides of the same coin. The same mechanism enabling depolarization-induced activation of the RyR1 in one direction was thought to facilitate Ca_V_1.1a channel gating in the reverse direction. In light of our present findings this model needs to be amended (Fig. 4), because in Ca_V_1.1e the skeletal muscle EC coupling mechanism is fully functional, but this does not facilitate gating of the Ca_V_1.1e channel. Apparently, retrograde coupling is only effective in a situation in which the calcium current is curtailed in the first place. Thus, in Ca_V_1.1a, where a mechanism depending on exon 29 curtails the calcium currents, the retrograde coupling mechanism may partially alleviate this limiting effect. In Ca_V_1.1e, however, where this limiting mechanism does not exist, retrograde coupling is no longer functional or necessary.

In this context it is important to note that the alleviating effect of retrograde coupling on the poor channel properties of Ca_V_1.1a is specific and incomplete. Specific, because it only augments the current amplitude but does not rescue the poor voltage sensitivity of Ca_V_1.1a. This is consistent with a recent report showing that dialysis of a 36 amino-acid peptide from the central domain of RyR1 into skeletal muscle fibers augmented Ca_V_1.1 current amplitude but not its voltage-dependence (20). Furthermore, the alleviation of current inhibition in Ca_V_1.1a is incomplete, because despite retrograde coupling Ca_V_1.1a current amplitudes are still severalfold lower than those of Ca_V_1.1e.

Nevertheless, augmentation of currents by retrograde coupling is somehow linked to the mechanism that limits current amplitude and voltage sensitivity of Ca_V_1.1a. From a structural point of view it is intriguing to consider how in Ca_V_1.1a the interaction of the RyR1 with the cytoplasmic loop between homologous domains II and III of the channel can counteract a mechanism that is dependent on the presence of an extracellular loop in domain IV encoded by exon 29. However, a recent study of Ca_V_1.2 voltage sensor movements demonstrated a strong allosteric coupling of the four repeats (21), suggesting that alternative splicing or altered protein-protein interactions in any one of the four voltage sensor domains might affect movement of the others as well. Along this line it is conceivable that exclusion of exon 29 might affect gating properties by altering interactions with auxiliary channel subunits. However, previously we have shown that Ca_V_1.1e was still sensitive to shRNA knockdown of *α*_2_*δ*-1 (4), and functional EC coupling implies intact interaction of Ca_V_1.1e with *β*_1a_ (22). Whether Ca_V_1.1e also interacts with the *γ*1 subunit, which has been shown to modify EC coupling as well as DHP binding (23), is still elusive.

### Ca_V_1.1e-SkLM reveals the contribution of calcium-induced calcium release in Ca_V_1.1e expressing muscle cells

Previously, we demonstrated that voltage-activated calcium transients in Ca_V_1.1e expressing myotubes are composed of a major current-independent component and a minor current-dependent component, which could be selectively blocked by Cd^2+^/La^3+^ (4). Thus, EC coupling in embryonic muscles, which predominantly express the Ca_V_1.1e variant, combines skeletal muscle and cardiac muscle EC coupling mechanisms. Here, we replaced the II-III loop of the Ca_V_1.1e splice variant with the corresponding loop of the Musca channel to specifically eliminate the skeletal muscle-type EC coupling component. Whereas the identical sequence substitution in Ca_V_1.1a fully disrupted skeletal muscle EC coupling (7), myotubes expressing Ca_V_1.1e-SkLM still displayed sizable calcium transients. The finding that these calcium transients were fully abolished when calcium currents were blocked with Cd^2+^/La^3+^, confirms that also in the context of Ca_V_1.1e the II-III loop substitution completely blocked skeletal muscle-type EC coupling. Furthermore, it demonstrated that the small calcium transients in myotubes expressing Ca_V_1.1e-SkLM were completely dependent on calcium influx through the mutated Ca_V_1.1e channel. Most likely the transients combine the calcium entering the myotubes through Ca_V_1.1e-SkLM plus calcium released from the SR by calcium-induced calcium release by RyR1. In agreement with previous studies (19), this shows that skeletal muscles are capable of cardiac muscle-type EC coupling if the channels conduct calcium. At depolarizations to +30 mV the fraction of this influx-dependent EC coupling component accounts for approximately one-quarter of the total Ca_V_1.1e calcium transients, whereas three quarters represent the current-independent direct EC coupling component. Therefore, in muscle cells expressing the embryonic Ca_V_1.1e splice variant, the calcium current-dependent component represents a substantial fraction of the total calcium signal in EC coupling.

### Ca_V_1.1e is sensitive to activation or block by DHPs

Given the fact that all Ca_V_1 channels studied so far are sensitive to DHPs and because the known binding site for DHPs (24) is not directly affected by skipping of exon 29, it was to be expected that also Ca_V_1.1e would be DHP sensitive. Nevertheless, the DHP sensitivity of the Ca_V_1.1e splice variant remained to be demonstrated. On the other hand, the II-III loop sequences also known to be responsible for retrograde coupling were not touched by skipping of exon 29, and still Ca_V_1.1e currents failed to be modulated by retrograde coupling. Moreover, the mode of current modulation by the DHP agonist Bay K 8644 (11) closely resembled the effects of exon 29 skipping, suggesting the possibility that both the channel agonist and the current-enhancing effect of deleting exon 29 might act on the same downstream gating mechanism. However, our pharmacological characterization of Ca_V_1.1e clearly demonstrated that the effects of Bay K 8644 and lack of exon 29 were additive, indicating that the deletion of exon 29 did not abolish the molecular mechanism of drug action. However, the magnitude of the Bay K 8644 effect was substantially lower compared to Ca_V_1.1a. This could either reflect a reduced Bay K 8644 sensitivity of the CaV1.1e splice variant or result from a saturation of the maximal conductance. Because Ca_V_1.1e channels already have a ∼10-fold higher open probability (4) the remaining dynamic range for pharmacological current enhancement may be limited compared to Ca_V_1.1a.

We further show that Ca_V_1.1e is also sensitive to the L-type channel antagonist nifedipine. This is important for the potential use of L-type channel blockers in the therapy of myotonic dystrophy. Tang et al. (9) demonstrated that in myotonic dystrophy type 1 and type 2 the splicing of Ca_V_1.1 was misregulated and that in muscles of adult human patients the degree of Ca_V_1.1e expression correlated with the severity of muscle weakness. Furthermore, forced skipping of exon 29 increased the number of central nuclei in muscles of a mouse model for myotonic dystrophy. Recently, it has been shown that expression of calcium regulating proteins and cytoplasmic calcium handling are dysregulated in cultured myotubes from myotonic dystrophy patients (10). Together, these findings strongly implicate altered calcium signaling and in particular overexpression of the embryonic splice variant Ca_V_1.1e in mature muscles in the pathophysiology of myotonic dystrophy. Thus, the use of clinically approved calcium channel blockers may be beneficial for patients suffering from myotonic dystrophy. Since in adults expression of Ca_V_1.1e is strongly increased in muscles affected by myotonic dystrophy the expected therapeutic effect would be specific for diseased muscle and because Ca_V_1.1e expression levels correlate with muscle weakness the effect would be greater the worse the disease. Whereas side effects on the virtually nonconducting adult skeletal muscle CaV1.1a splice variant would not be an issue, a confounding factor will be the known effects of these drugs on cardiovascular function. Our finding that the IC50 of nifedipine action on Ca_V_1.1e (0.37 *μ*M) is in the same range as that reported for the Ca_V_1.2, the major target for DHPs in smooth muscle cells, indicates that at the therapeutic dose side effects like hypotension would be expected.

## Conclusions

In conclusion, our biophysical analysis of the embryonic splice variant Ca_V_1.1e indicates that Ca_V_1.1e LTCC are not subject to retrograde coupling with the RyR1 and that the physiological current modulation of Ca_V_1.1a by retrograde coupling converges on the same molecular mechanism by which inclusion of the sequences encoded by exon 29 limits the size of the calcium current. On the other hand, our pharmacological characterization of Ca_V_1.1e demonstrates that the channel agonist Bay K 8644 and inclusion of exon 29 use distinct molecular mechanisms for current enhancement. Its sensitivity to DHP calcium channel blockers identifies Ca_V_1.1e as a potential drug target for the treatment of myotonic dystrophy.

## Figures and Tables

**Table 1:** Properties of adult and embryonic Ca_V_1.1 currents

	Ca_V_1.1a				Ca_V_1.1e			
	Control (*n* = 15)	Bay K 0.1 *μ*M (*n* = 3)	Bay K 1 *μ*M (*n* = 8)	Bay K 10 *μ*M (*n* = 7)	Control (*n* = 15)	Bay K 0.1 *μ*M (*n* = 4)	Bay K 1 *μ*M (*n* = 8)	Bay K 10 *μ*M (*n* = 7)
I_peak_	−2.0 ± 0.2 pA/pF	1.8 ± 0.1	3.7 ± 0.4	4.4 ± 0.6	−10.4 ± 0.5 pA/pF	1.5 ± 0.2	2.0 ± 0.1	2.0 ± 0.2
V_1/2_ (mV)	35.1 ± 1.7	−15.0 ± 6.2	−7.4 ± 1.2	−19.1 ± 1.5	6.4 ± 1.8	−10.8 ± 5.4	−12.5 ± 1.8	−9.3 ± 2.6

All data are presented as mean ± SE of the mean. I_peak_ values with BayK are presented as fold increase compared to the control.V_1/2_ values with BayK are expressed as voltage shifts from the control.

**Table 2:** Nifedipine effect on embryonic Ca_V_1.1e currents

Nifedipine	–	0,1 *μ*M	0,5 *μ*M	5 *μ*M
I_peak_ (pA/pF)	14.7 ± 2.0	11.9 ± 2.0	6.5 ± 2.0	1.4 ± 0.3
V_1/2_ (mV)	2.7 ± 1.2	–	4.3 ± 2.2	–
*n*	12	3	5	4

All data are presented as mean ± SE of the mean.
